# Identification of CD24 as a potential diagnostic and therapeutic target for malignant pleural mesothelioma

**DOI:** 10.1038/s41420-020-00364-1

**Published:** 2020-11-18

**Authors:** Sivasundaram Karnan, Akinobu Ota, Hideki Murakami, Md Lutfur Rahman, Muhammad Nazmul Hasan, Md Wahiduzzaman, Ichiro Hanamura, Lam Quang Vu, Akihito Inoko, Toshinori Hyodo, Hiroyuki Konishi, Shinobu Tsuzuki, Yoshitaka Hosokawa

**Affiliations:** 1 Department of Biochemistry, Aichi Medical University School of Medicine, Nagakute, Aichi Japan; 2 Department of Pathology, Aichi Medical University School of Medicine, Nagakute, Aichi Japan; 3 Division of Hematology, Department of Internal Medicine, Aichi Medical University School of Medicine, Nagakute, Aichi Japan

**Keywords:** Mesothelioma, Targeted therapies

## Abstract

Malignant pleural mesothelioma (MPM) is an aggressive malignancy of the pleura that is currently incurable due to the lack of an effective early diagnostic method and specific medication. The *CDKN2A* (*p16*) and *NF2* genes are both frequently mutated in MPM. To understand how these mutations contribute to MPM tumor growth, we generated NF2/p16 double-knockout (DKO) cell clones using human MeT-5A and HOMC-B1 mesothelial cell lines. Cell growth and migration activities were significantly increased in DKO compared with parental cells. cDNA microarray analysis revealed differences in global gene expression profiles between DKO and parental cells. Quantitative PCR and western blot analyses showed upregulation of CD24 concomitant with increased phosphorylation of AKT, p70S6K, and c-Jun in DKO clones. This upregulation was abrogated by exogenous expression of NF2 and p16. *CD24* knockdown in DKO cells significantly decreased TGF-β1 expression and increased expression of E-cadherin, an epithelial–mesenchymal transition marker. CD24 was highly expressed in human mesothelioma tissues (28/45 cases, 62%) and associated with the loss of NF2 and p16. Public data analysis revealed a significantly shorter survival time in MPM patients with high *CD24* gene expression levels. These results strongly indicate the potential use of CD24 as a prognostic marker as well as a novel diagnostic and therapeutic target for MPM.

## Introduction

Malignant pleural mesothelioma (MPM) is an aggressive malignancy of the pleura that is associated with asbestos exposure after 30–40 years of latency^1,2^. Patients with MPM are usually diagnosed at an advanced stage of the disease and their prognosis remains poor. The median survival after diagnosis is 6–12 months and the standard treatment agents, pemetrexed and cisplatin, are relatively ineffective at increasing survival time^2,3^. Despite the restricted and banned use of asbestos, MPM is increasingly being diagnosed in young individuals and women^4,5^. Other risk factors, including exposure to erionite fibers, therapeutic ionizing radiation to the chest, and germline *BRCA1-associated protein 1* (*BAP1*)-inactivating mutations, have been causally linked to MPM^1^. Therefore, new therapies based on improving patient survival are still required^6,7^.

Molecular biological studies in MPM have revealed frequent genetic alterations of tumor suppressor genes, including *neurofibromatosis 2* (*NF2*), *cyclin-dependent kinase inhibitor 2A* (*p16*), and *BAP1*^8–15^. Furthermore, multiplex molecular analyses with whole-exome sequencing, high-density array comparative genomic hybridization, and immunohistochemistry (IHC) have disclosed that somatic *BAP1* mutations and deletions were present in >60% of MPM patients^16,17^. We recently reported that fibroblast growth factor receptor 2 is highly expressed in NF2-knockout mesothelial cell lines and is a candidate molecule for the development of therapeutic and diagnostic strategies targeting MPM^18^. A previous study using NF2 and p16 double-knockout (DKO) mice indicated that these genes, when mutated, contribute to mesothelioma development^19^. However, it remains unclear how these complex mutations contribute to tumor formation in MPM. We established NF2/p16-DKO cell clones in human immortalized mesothelial cell lines and identified several genes regulated by NF2 and p16.

CD24 is a glycosylphosphatidylinositol-linked sialoprotein that was shown to be present in the cell membranes of B lymphocyte precursors, neutrophils, neuronal cells, and some epithelial cells, and is highly expressed in several types of cancers^20–30^. It was also shown to be an independent prognostic marker of reduced patient survival in ovarian cancer and non-small cell lung cancer^21,22^. Furthermore, CD24 is reportedly related to epithelial–mesenchymal transition (EMT) in ovarian and pancreatic cancers^31,32^. The present study found that CD24 is highly expressed in NF2/16-DKO cell clones and human MPM tissues, highlighting it as a potential diagnostic and therapeutic target for MPM.

## Results

### NF2/p16 DKO enhances cell proliferation, colony formation, and migration in MeT-5A and HOMC-B1 human mesothelial cell lines

**Fig. 1:**
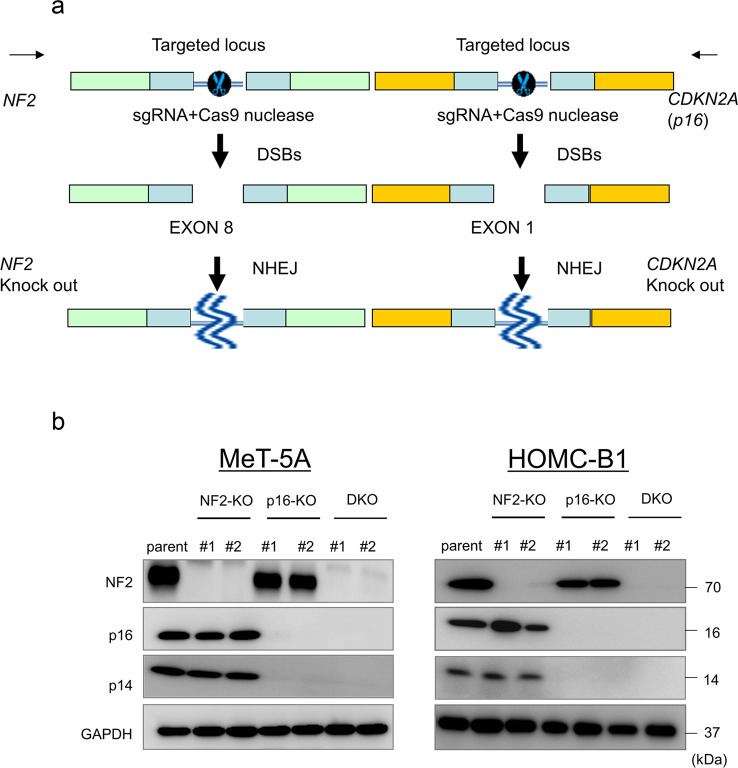
Generation of NF2-knockout (*NF2*-KO), CDKN2A-knockout (p16-KO), and NF2/p16 double-knockout (*NF2/p16*-DKO) cell clones using a CRISPR/Cas9 system with the MeT-5A and HOMC-B1 human mesothelial cell lines. **a** Single-guide RNA sequences were designed against exon 8 of the *NF2* gene (left side) and exon 1 of the *CDKN2A* (*p16*) gene (right side). **b** Independent NF2-knockout clones (NF2-KO#1 and #2), p16-knockout clones (p16-KO#1 and #2), and NF2/p16-DKO clones (DKO#1 and #2) were established. NF2 and p16/14 protein expression was determined by western blot analysis. GAPDH was used as an internal standard.

**Fig. 2:**
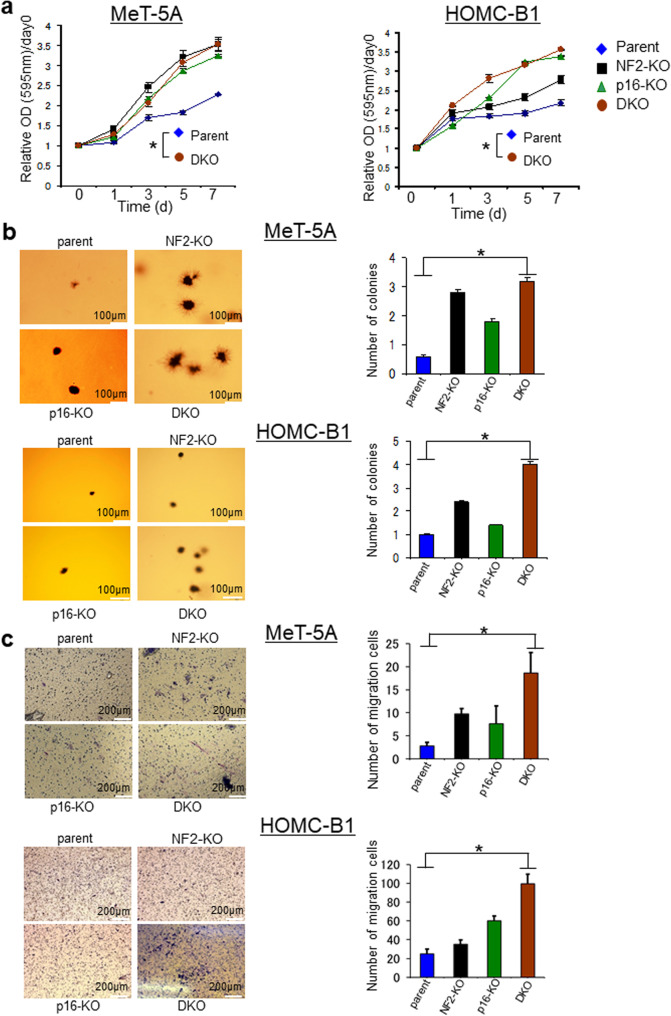
Cellular phenotype of parent, NF2-KO, p16-KO, and NF2/p16-DKO-Met-5A and HOMC-B1 cells. **a** MTT analysis of the growth rate in parental cells, NF2-KO cell clones, p16-KO cell clones, and NF2/p16-DKO cell clones. Data are mean ± SEM (*n* = 3). *P < 0.05, statistically significant difference between parental and DKO cells. **b** Representative soft agar colony formation assays are shown. Right bar graphs represent the number of stained colonies. Scale bar = 100 μm. Data represent mean ± SEM (*n* = 3). *P < 0.05, statistically significant difference. **c** Representative migration assays using a Boyden chamber are shown. The right bar graph represents the number of stained colonies. Scale bar = 200 μm. Data represent mean ± SEM (*n* = 3). Asterisks (*) indicate statistically significant differences (*P < 0.05).

To investigate the roles of *NF2* and *p16* in mesothelial cells, we established two NF2-knockout clones (NF2-KO#1 and #2), two p16-knockout clones (p16-KO#1 and #2), and two NF2/p16-DKO cell clones (DKO #1 and #2) using MeT-5A and HOMC-B1 human mesothelial cell lines with the CRISPR/Cas9 system (Fig. 1a). Protein expression of NF2 and p16 was not detected in DKO #1 and #2 (Fig. 1b). Also, no p14ARF expression, an alternative form of p16, was detected after targeting exon 1 of p16 (Fig. 1b). We examined cell proliferation of the established clones using MTT (3-(4,5-dimethylthiazol-2-yl)-2,5-diphenyltetrazolium bromide) assay and found that the cell growth ratio was significantly increased in the DKO clones compared with that in the parental cells (Fig. 2a). The DKO clones also formed more colonies in soft agar compared with the parental cells (Fig. 2b). Furthermore, migration activity was significantly higher in the DKO clones (Fig. 2c). These results indicate that NF2/p16 inactivation enhances cell growth, clonogenicity, and migration in the mesothelial cells.

### Gene expression change induced by disruption of NF2/p16

**Fig. 3:**
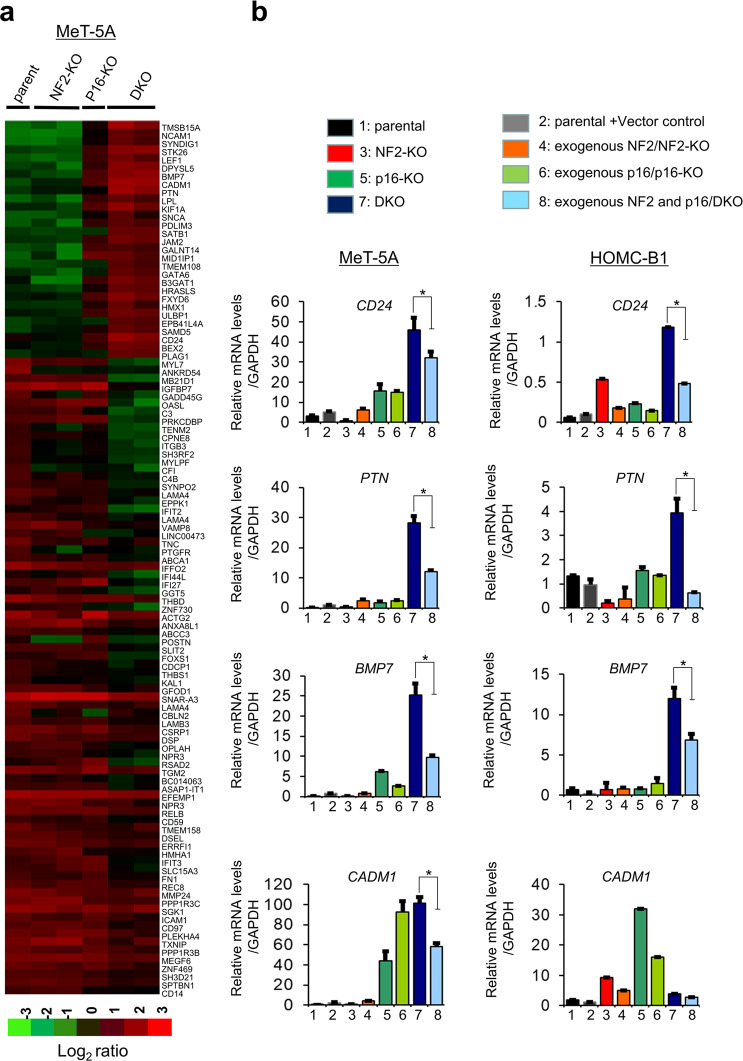
Gene expression analysis. Parental MeT-5A cells and knockout cell clones were seeded in 6-well plates and incubated for 24 h. Total RNA was extracted and cDNA microarray analysis was performed using Agilent Whole Human Genome cDNA Microarray Kit (4 × 44 K; Design ID, 026652). **a** Heat map showing upregulated genes (29 genes; fold change > 20.0) and downregulated genes (76 genes, fold change < 0.05) in the DKO cell clones compared with the parent cells, NF2-KO cell clones, and p16-KO cell clones. The heat map was constructed using normalized values of each sample with Tree View (Cluster 3.0) software. Corresponding upregulated or downregulated genes in the heat map are shown on the right side. **b** Quantitative real-time PCR (qRT-PCR) analysis. Four genes that were upregulated in DKO cell, as detected using cDNA microarray analysis, were selected for qRT-PCR analysis using the SYBR green method. Relative gene expression levels are shown after normalization to *GAPDH* mRNA expression. Mean values were compared with the normal control value to calculate the relative amounts of transcripts. Data represent mean ± SEM (*n* = 3). Asterisks (*) indicate statistically significant differences (*P < 0.05).

To identify genes involved in enhanced cell growth and migration, we performed comprehensive cDNA microarray analysis in the DKO, NF2-KO, and p16-KO clones, as well as the parental cells. To compare gene expression profiles, normalized values of raw microarray data were calculated and clustered according to differential gene expression. Expression of 29 genes was upregulated >20.0-fold (Supplementary Table S3) and 76 genes were downregulated <0.05-fold (Supplementary Table S4). Clustering of the 105 genes showed a distinct gene expression pattern among the DKO, NF2-KO, and p16/p14-KO clones, and the parental cells (Fig. 3a). To further confirm the effect of NF2/p16 on changes to gene expression, we generated clones exogenously expressing NF2 in the NF2-KO clone (exogenous NF2/NF2-KO), exogenous p16 in the p16-KO clone (exogenous p16/p16-KO), exogenous NF2 and p16 in the DKO clone (exogenous NF2 and p16/DKO). We then performed quantitative real-time PCR (qRT-PCR) analysis for four cell surface receptor genes (*PTN*, *CD24*, *BMP7*, and *CADM1*), because their protein products are easily detectable by molecular diagnosis and are also related to cell survival, proliferation, or tumor growth. The qRT-PCR results revealed increased expression of *PTN*, *CD24*, *BMP7*, and *CADM1* genes in the DKO clones compared with the parental cells and NF2-KO and p16-KO clones (Fig. 3b). Furthermore, increased expression of *PTN*, *CD24*, and *BMP7* was abrogated in the DKO clones exogenously expressing NF2 and p16 (exogenous NF2 and p16/DKO) (Fig. 3b), strongly suggesting that the affected gene products were downstream of NF2 and p16 signaling.

### Effect of NF2/p16 DKO on CD24 expression and cell cycle-related molecules

**Fig. 4:**
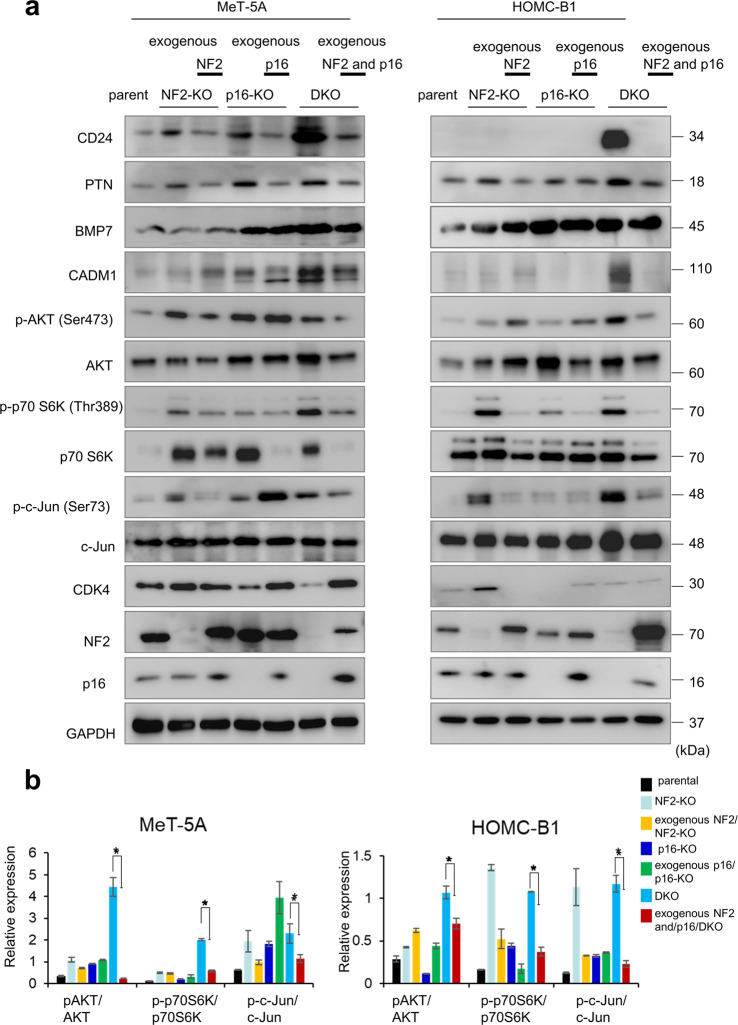
Protein expression analyses. **a** Western blot analysis showing expression of the indicated proteins in parent cells, NF2-KO cell clones, exogenous NF2 expression/NF2-KO cell clones, p16-KO cell clones, exogenous p16 expression/p16-KO cell clones, DKO cell clones, and exogenous NF2 and p16 expression/DKO cell clones. Cell lysates obtained were used for western blotting analysis to detect protein levels using the specific antibodies listed in Supplementary Table S1. The cell lysates obtained were used for western blotting analysis. GAPDH was used as an internal control. **b** Quantitation of p-AKT, p70S6K, and c-Jun in MeT-5A and HOMC-B1 cells. p-AKT, p70S6K, and c-Jun expression levels were quantified by measuring the ratio between phosphorylated and total levels. Data are mean ± SEM (*n* = 3). *P < 0.05, statistically significant difference.

CD24 has been implicated in the pathogenesis of a wide range of human cancers and emerged as a novel anticancer application in a panel of solid cancers^33–36^. Therefore, we focused on CD24 as its expression was increased in DKO cells (Fig. 4a). Exogenous expression of NF2 and p16 resulted in reduced CD24 expression in NF2 and p16/DKO clones (Fig. 4a), indicating that CD24 expression is negatively regulated by NF2/p16 signaling in mesothelial cells.

Next, the phosphorylation levels of AKT, p70S6K, and c-Jun were investigated and quantified to determine cell growth signaling. As shown in Fig. 4a, b, we observed increased phosphorylation of cellular growth and proliferation-related signaling molecules, including AKT, p70S6K, and c-Jun, in the DKO clones. PTN, BMP7, and CADM1 protein products, as well as CD24, were increased in the DKO clones (Fig. 4a). To confirm the effect of NF2 and p16 on the expression of these signaling proteins, we utilized DKO clones exogenously expressing NF2 and p16 (exogenous NF2 and p16/DKO) (Fig. 4a). In the exogenous NF2 and p16/DKO clones, increased expression of CD24, PTN, BMP7, CADM1, p-pAKT, p-p70S6K, and p-c-Jun was downregulated, strongly suggesting that these proteins function downstream of NF2 and p16 signaling. Different expression patterns between *CADM1* mRNA and its protein were observed in HOMC-B1 cells. Different expression patterns of PTN and BMP7 proteins from their mRNAs in MeT-5A and HOMC-B1 cells were also observed. The difference would be, in part, due to a post-transcriptional modification.

### *CD24* knockdown downregulates TGF-β1 and cell proliferation in the absence of *NF2*/*p16* gene expression

**Fig. 5:**
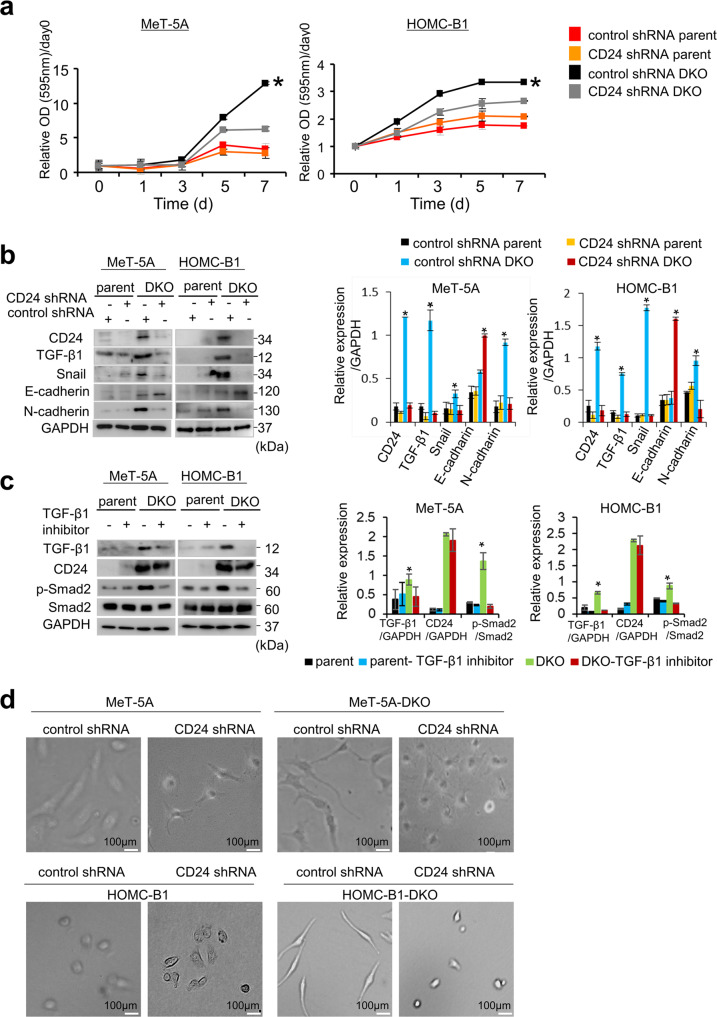
*CD24* knockdown reduces cell proliferation and induces gene expression and morphological changes of EMT phenotype in DKO cells. **a** Effect of CD24 shRNA on cell proliferation. DKO cells (Met-5A and HOMC-B1) and parent cells were transfected with CD24 shRNA and control shRNA vectors. **b** Effect of *CD24* shRNA on protein expression. **c** Effect of TGF-β1 inhibitor (vactosertib) on CD24 expression. Cells were treated with 2.5 μM vactosertib for 24 h and cell lysates were used for western blot analysis. GAPDH or total protein was used as an internal control. **d** Effect of *CD24* shRNA on morphological changes in DKO cells and parent cells. DKO cells and parent cells were transfected with *CD24* shRNA and control shRNA vectors. After 48 h of incubation, photomicrographs were taken depicting cell morphological in control shRNA-parent, *CD24* shRNA-parent cells, control shRNA-DKO cells, and *CD24* shRNA-DKO cells. Scale bar = 100 μm. All data are mean ± SEM (*n* = 3). *P < 0.05, statistically significant difference.

To investigate the involvement of CD24 in cell proliferation, we knocked down *CD24* in the DKO clones and parental cells. MTT assay showed that cell growth was significantly decreased in the CD24 shRNA-DKO cells compared with the DKO clones (Fig. 5a). Furthermore, colony formation also decreased in the CD24 shRNA-DKO cells (Supplementary Fig. S1). These results strongly suggest that CD24 may play an important role in cell growth and clonogenicity of mesothelioma cells with loss of p16 and NF2 expression.

In DKO cell clones, we observed gene expression changes characteristic of EMT phenotypes, such as increased expression of transforming growth factor-β1 (TGF-β1) (as an inducer of EMT phenotypes), and Snail and N-cadherin (as mesenchymal markers). Notably, *CD24* knockdown decreased expression of TGF-β1, Snail, and N-cadherin, and increased expression of E-cadherin in DKO clones (Fig. 5b), strongly suggesting that CD24 may be related to the EMT phenotype. The TGF-β1 inhibitor, vactosertib, did not affect expression of CD24 in DKO clones, implying that CD24 functions upstream of TGF-β1 expression (Fig. 5c). Increased phosphorylation of SMAD2 in DKO cells compared to parental cells and subsequent decrease after treatment with the inhibitor were also observed (Fig. 5c). Furthermore, DKO clones with higher CD24 expression showed a spindle cell morphology, which is a morphological feature of EMT (Fig. 5d). Knockdown of *CD24* also led to a change in cell morphology from spindle cell to circle shape, as well as an increased number of cell–cell contacts. To obtain further experimental evidence for the EMT phenotype, immunofluorescence analyses were performed to examine the expression of desmoplakin, one of the desmosomes^37^. A loss of desmoplakin expression in DKO cells and a mesothelioma cell line compared to mesothelial cells was found (Supplementary Fig. S2). Taken together, these results prompted us to speculate that CD24 induces the EMT phenotype in mesothelial cells with loss of NF2 and p16.

### IHC analysis of CD24 expression in patients with MPM

**Fig. 6:**
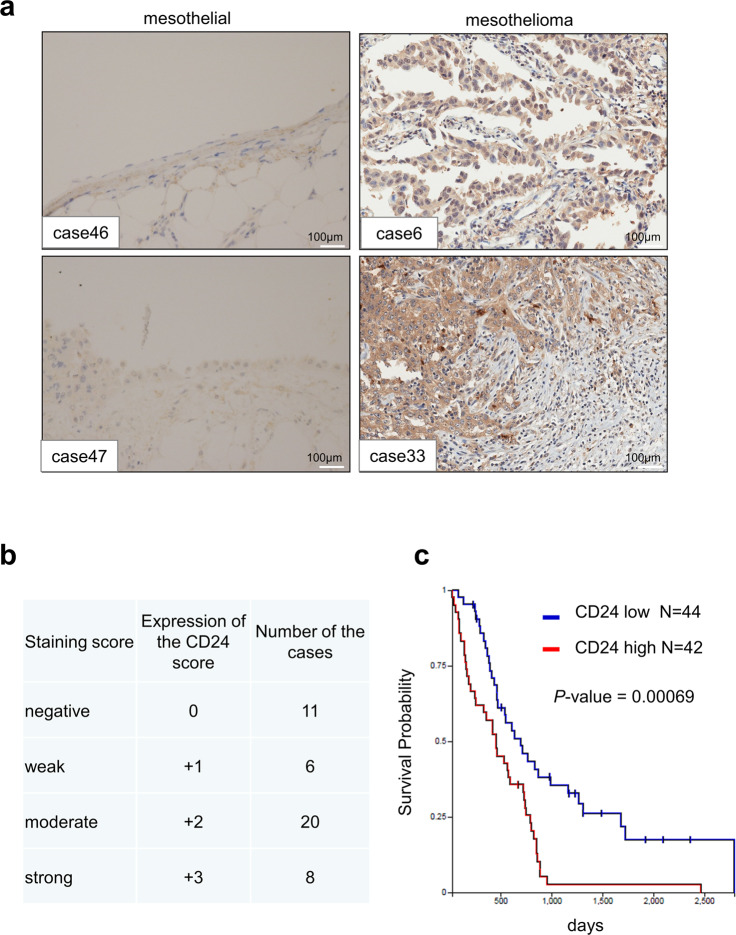
Immunohistochemical (IHC) analysis of CD24 expression. **a** Representative IHC results showing CD24 expression in two MPM tissues (right panels, cases 6 and 33) and two normal mesothelium tissues (left panels, cases 46 and 47). **b** Summary of IHC results in MPM tissues. Immunoreactivity was independently evaluated by two investigators (S.K. and H.M.). Staining intensity was scored as strong (3+), moderate (2+), weak (1+), or negative (0). The number of cases and their staining intensities are shown in the right panel. **c** Kaplan–Meier analysis was conducted to assess the value of CD24 in overall survival of MPM patients in TCGA mesothelioma obtained from the UCSC Xena database. Fluorescence values above the median were considered high *CD24* expression, whereas fluorescence values below the median were considered low *CD24* expression. Scale bar = 100 μm.

To investigate the role of CD24 in human MPMs, we performed immunohistochemical analysis to examine protein expression levels of CD24 in 45 MPMs and three normal mesothelium tissue samples (Table 1 and Fig. 6a). Microscopic analysis detected 8 strong (3+), 20 moderate (2+), and 6 weak (1+) CD24-positive signals among 45 MPM tissue samples (Fig. 6b), whereas CD24 expression was not detectable in three normal mesothelium tissues (Table 1 and Fig. 6a, left panels). Also, the CD24 expression pattern in three subtypes (epithelioid, sarcomatous, and biphasic)^1^ is summarized in Table S5. A high frequency of CD24 expression was observed in the epithelioid type. To further validate *CD24* expression in MPM patients, we analyzed a public cohort of 86 patients from The Cancer Genome Atlas Mesothelioma. Our analysis revealed that overall survival in MPM patients with higher *CD24* expression levels was shorter than that in those with low *CD24* expression levels (Fig. 6c). According to the log_2_ value of CD24 probe, patients were divided into two groups: high (log_2_ value > 6.938, *n* = 42) and low (log_2_ value < 6.938, *n* = 44) *CD24* expression. Thus, high *CD24* expression was significantly associated with overall survival (*P* < 0.0001).

**Table 1:** Summary of immunohistochemistry in this study.

Case nos.	Age	Sex	Organ (anatomic site)	Pathology diagnosis	Stage	CD24 intensity	Proportion (%)
1	64	M	Thoracic cavity	Malignant mesothelioma of chest wall	I	1+	100
2	48	M	Thoracic cavity	Malignant mesothelioma of right pleura	I	0	-
3	58	F	Thoracic cavity	Malignant mesothelioma of pleura	I	2+	100
4	49	F	Thoracic cavity	Malignant mesothelioma of pleura	I	2+	60
5	22	M	Thoracic cavity	Malignant mesothelioma of pleura	I	1+	60
6	49	M	Thoracic cavity	Malignant mesothelioma of pleura	I	3+	100
7	32	M	Thoracic cavity	Malignant mesothelioma of pleura	I	2+	100
8	29	M	Thoracic cavity	Malignant mesothelioma of pleura	I	2+	60
9	31	F	Thoracic cavity	Malignant mesothelioma of thoracic cavity	I	2+	100
10	70	F	Thoracic cavity	Malignant mesothelioma of right pleura	II	2+	100
11	47	M	Thoracic cavity	Malignant mesothelioma of left pleura	II	0	-
12	60	M	Thoracic cavity	Malignant mesothelioma of pleura	II	3+	100
13	46	M	Thoracic cavity	Malignant mesothelioma of thoracic cavity	II	2+	100
14	35	M	Thoracic cavity	Malignant mesothelioma of pleura	II	0	-
15	83	M	Thoracic cavity	Malignant mesothelioma of pleura	III	0	-
16	56	M	Thoracic cavity	Malignant mesothelioma of pleura mediastinalis	III	0	-
17	67	F	Thoracic cavity	Malignant mesothelioma of right thoracic cavity	IV	3+	100
18	57	M	Abdominal cavity	Malignant mesothelioma of peritoneum	I	3+	100
19	29	M	Abdominal cavity	Malignant mesothelioma of peritoneum	I	0	-
20	71	M	Abdominal cavity	Malignant mesothelioma of mesostenium	II	2+	100
21	63	F	Abdominal cavity	Malignant mesothelioma of peritoneum	II	2+	100
22	48	F	Abdominal cavity	Malignant mesothelioma of peritoneum	II	2+	100
23	60	F	Abdominal cavity	Malignant mesothelioma of peritoneum	II	2+	100
24	60	F	Abdominal cavity	Malignant mesothelioma of peritoneum	II	0	-
25	47	M	Abdominal cavity	Malignant mesothelioma of peritoneum	II	2+	100
26	33	F	Abdominal cavity	Malignant mesothelioma of peritoneum	II	1+	100
27	76	F	Abdominal cavity	Malignant mesothelioma of hypogastrium	II	3+	100
28	78	M	Abdominal cavity	Malignant mesothelioma of hypogastrium	II	1+	100
29	41	F	Abdominal cavity	Malignant mesothelioma of hypogastrium	II	0	-
30	28	M	Abdominal cavity	Malignant mesothelioma of left retroperitoneum	II	2+	100
31	5	F	Abdominal cavity	Malignant mesothelioma of right superior belly	II	2+	100
32	45	M	Abdominal cavity	Malignant mesothelioma of peritoneum	II	1+	100
33	69	M	Abdominal cavity	Malignant mesothelioma of peritoneum	II	3+	100
34	78	M	Abdominal cavity	Malignant mesothelioma of hypogastrium	III	2+	100
35	33	M	Abdominal cavity	Malignant mesothelioma of retroperitoneum	III	0	-
36	5	F	Abdominal cavity	Malignant mesothelioma of abdominal cavity	III	1+	100
37	40	F	Heart	Malignant mesothelioma of left cardiac atrium	II	0	-
38	50	F	Heart	Malignant mesothelioma of left cardiac atrium	II	2+	100
39	43	F	Heart	Malignant mesothelioma of pericardium	II	2+	100
40	43	F	Heart	Malignant mesothelioma of pericardium	I	2+	100
41	50	F	Heart	Malignant mesothelioma of pericardium	I	2+	100
42	44	F	Colon	Malignant mesothelioma of tunica serosa coli	II	0	-
43	56	F	Colon	Malignant mesothelioma of tunica serosa coli	II	2+	100
44	60	M	Epoploon	Malignant mesothelioma of epoploon	II	3+	100
45	60	M	Epoploon	Malignant mesothelioma of epoploon	II	3+	100
46	67	M	Thoracic cavity	Parietal pleura		Normal	-
47	87	F	Thoracic cavity	Parietal pleura		Normal	-
48	60	M	Thoracic cavity	Parietal pleura		Normal	-

Each intensity of the positive signal for CD24 was evaluated by two investigators.

Furthermore, NF2 and p16 expression, together with CD24 expression in human MPM tissue arrays, was immunohistochemically examined. CD24-positive expression showed 20 (100%) of 20 cases with both NF2- and p16-negative expression, whereas CD24-positive expression showed 8 (42%) of 19 cases with both NF2- and p16-positive expression (Supplementary Table S6 and Supplementary Fig. S3). These results suggest that CD24-positive expression might be associated with the loss of NF2 and p16 expression in MPM tissues.

## Discussion

*NF2*, *p16*, and *BAP1* are the three major tumor suppressor genes that frequently undergo genomic alterations in MPMs. In the present study, we generated both NF2 and p16-knockout isogenic clones using the human normal mesothelial cell lines, MeT-5A and HOMC-B1, and showed that loss of *NF2* and *p16* expression enhanced cell growth, clonogenicity, and migration activities with global changes in gene expression. Our findings strongly suggest that CD24 expression occurs downstream of NF2 and p16 in mesothelial cells and is correlated with overall survival of MPM patients.

CD24 is frequently overexpressed in several types of solid tumors and is correlated with poor prognosis^22,25,28,38,39^. Thus, CD24 could be a potential target for a monoclonal antibody-mediated therapy. Antibody-mediated therapy toward cell surface antigens, such as CD20, has been used as treatment for non-Hodgkin’s lymphoma^40,41^. Therefore, the present study focused on CD24, as its expression is increased in DKO clones. Knockdown of *CD24* reduced cell growth and clonogenicity. CD24 was also reported to be related to EMT, which is an important step leading to invasion, migration, and resistance to apoptosis of various cancer cells^28,31,32^. EMT phenotypes are characterized by decreased expression of E-cadherin as an epithelial marker, and increased expression of TGF-β1 as an inducer of EMT and Snail, Twist, Slug, vimentin, and N-cadherin as mesenchymal markers, and are responsible for poor prognosis in patients with various cancers^42^. In the present study, knockdown of *CD24* decreased the expression of TGF-β1, Snail, and N-cadherin, and increased the expression of E-cadherin in DKO clones, strongly suggesting that CD24 may contribute to the EMT phenotype in the mesothelial cells. To further explore the relationship between CD24 and TGF-β1, we examined the effect of TGF-β1 inhibitor in the DKO cells. Knockdown of *CD24* decreased the expression of TGF-β1; however, TGF-β1 inhibitor did not alter the expression of CD24, indicating that CD24 functions upstream of TGF-β1. In addition, DKO clones with higher expression of CD24 showed a spindle cell morphology, which is a morphological feature of EMT. We also observed that knockdown of *CD24* changed the morphology of spindle cells to circles. Thus, our results suggest that CD24 contributes to EMT phenotypes in mesothelial cells. Our results indicate that CD24 expression is closely associated with changes in both gene expression and cell morphology characteristic of EMT phenotypes. Desmosome is a cell structure specialized for cell-to-cell adhesion^37^. The loss of expression of many desmosome proteins has been reported in different cancers and may contribute to EMT^43,44^. Thus, the expression of desmoplakin, one of the desmosomes, was analyzed, and loss of desmoplakin expression was found in DKO cells and mesothelioma cell lines, providing further experimental evidence for the EMT phenotype^45^; therefore, we propose that CD24 expression induces the EMT phenotypes involved in the pathogenesis of MPM.

IHC analysis showed that CD24 was commonly expressed in 62% of human MPM tissues. Furthermore, analysis of public data revealed that MPM patients with higher expression of the *CD24* gene showed a significantly shorter survival time. In addition, loss of *NF2* and *p16* resulted in increased expression of CD24, and subsequent rescue of the two genes decreased its expression in the MeT-5A and HOMC-B1 cell lines. More importantly, CD24 expression was associated with the loss of NF2 and p16 expression in human MPM tissues. These results indicate that CD24 expression is intimately regulated by NF2 and p16 expression. Furthermore, knockdown of *CD24* in the DKO clones led to retardation of cell growth and colony formation, strongly suggesting that CD24 may play a pivotal role in the proliferation and clonogenicity of DKO cells.

In the present study, global gene expression changes in response to loss of *NF2* and *p16* in the MeT-5A and HOMC-B1 human mesothelial cell lines were demonstrated. CRISPR/Cas9-mediated loss of *NF2* and *p16* enhanced cell proliferation and CD24 expression, and disruption of *CD24* reduced the proliferation of the cells. Although the molecular mechanisms underlying the upregulation of CD24 via loss of *NF2* and *p16* remain unclear, CD24 was commonly expressed in MPM tissues and was associated with poor prognosis of MPM patients. Therefore, CD24 may be used as a prognostic marker as well as a novel diagnostic and therapeutic target for MPM. Further studies are warranted to develop a new therapeutic approach for the treatment of MPM.

## Materials and methods

### Cell culture

The immortalized normal human mesothelial cell lines, MeT-5A (pleural mesothelial) and HOMC-B1 (omental mesothelial; epithelioid type), and mesothelioma cell lines (Y-MESO-9) were kindly provided by Dr. Y. Sekido at the Division of Molecular Oncology, Aichi Cancer Center Research Institute, Nagoya, Japan. The HOMC-B1 cell line was maintained as described previously^46^. The MeT-5A was maintained in RPMI-1640 (Wako, Osaka, Japan) medium containing 10% fetal bovine serum (Sigma) and 1% penicillin/streptomycin (Wako) at 37 °C in a 5% CO_2_ humidified atmosphere.

### Gene knockout using the clustered regularly interspaced short palindromic repeat (CRISPR)/Cas9 system

The CRISPR/Cas9 system was used to disrupt expression of the *NF2* and *CDKN2A*(*p16*) genes as described previously^47^. pSpCas9(BB)-2A-GFP (PX458) was a gift from Feng Zhang (Addgene plasmid #48138)^47^. In brief, a single-guide RNA (sgRNA) sequence was selected using an optimized CRISPR design (http://crispr.mit.edu/). The sgRNA sequence used for *NF2* was 5′-AAACATCTCGTACAGTGACA-3′ and the sequence used for *p16* was 5′-ACCGTAACTATTCGGTGCGT-3′, corresponding to exons 8 and 1, respectively. Plasmids expressing hCas9 and the sgRNA were prepared by ligating oligonucleotides into the *Bbs*I site of PX458 (*NF2*/PX458 and *p16*/PX458). Knockout clones were established by electroporation of 1 μg of *NF2*/PX458 or *p16*/PX458 plasmid into 1 × 10^6^ cells using a 4D-Nucleofector system instrument (Lonza Japan, Tokyo, Japan). Three days post transfection, cells expressing green fluorescent protein were sorted using BD FACSARIA III (BD bioscience). A single clone was selected, expanded, and used for biological assays.

### Construction of RNAi vectors and expression vectors

RNA interference vectors were constructed by inserting shRNA oligonucleotides into the pLentiLox3.7 plasmid (Addgene) under the control of the U6 promoter. One shRNA oligonucleotide was designed for the target sequence of the hairpin loop of *CD24* (sh, 5′-TGCATTGACCACGACTAA-3′). A control shRNA vector was also constructed using a scrambled (scr) *CD24* sequence (scr, 5′-GGATAAACTAAGGGATAGGAA-3′). DKO and parent cells (1 × 10^6^ cells) were nucleofected with 1 μg of each vector using a 4D-Nucleofector instrument (Lonza Japan). After 48 h of incubation, cell lysates were prepared and used for western blot analysis.

The NF2/pcDNA3.1 vector was transfected into NF2-KO clones, the p16/pcDNA3.1 vector was transfected into p16-KO clones, and the NF2/pcDNA3.1 and p16/pcDNA3.1 vectors were transfected into DKO clones using the 4D-Nucleofector System. After transfection, cells were incubated for 48 h, washed with phosphate-buffered saline (PBS), and lysed in loading buffer. The lysates were used for Western blot analysis.

### Quantitative real-time PCR

qRT-PCR analysis for *PTN*, *CD24*, *BMP7*, and *CADM1* was performed using SYBR Green I as previously described^48^. Glyceraldehyde 3-phosphate dehydrogenase was used as an internal control. The sequences of the primers for *PTN*, *CD24*, *BMP7*, and *CADM1* used in this study are summarized in Supplementary Table S1.

### cDNA microarray analysis

cDNA microarray analysis was performed according to the manufacturer’s instructions (Agilent Technologies). In brief, cDNA synthesis and cRNA labeling with cyanine 3 (Cy3) dye were performed using the Agilent Low Input Quick Amp Labeling Kit (Agilent Technologies). Cy3-labeled cRNA was purified, fragmented, and hybridized on a Human Gene Expression 4 × 44K v2 Microarray Chip containing 27,958 Entrez Gene RNAs using a Gene Expression Hybridization kit (Agilent Technologies). Raw and normalized microarray data were submitted to the Gene Expression Omnibus database at the National Center for Biotechnology Information (accession number GSE116000; https://www.ncbi.nlm.nih.gov/geo/query/acc.cgi?acc=GSE116000). Gene set enrichment analysis was performed according to the instructions.

### Cell growth assay

Cell growth rate was determined using MTT assay. Briefly, cells (1 × 10^3^ per well) were seeded into 96-well plates and cultured for the indicated times. Subsequently, 10 μL of MTT solution (5 mg/mL; Sigma-Aldrich) was added to each well and cells were further incubated for 4 h. Next, cell lysis buffer was added to the wells to dissolve the colored formazan crystals produced by MTT. The relative optical density (OD) at 595 nm was calculated by dividing the OD on day 0 at each time point (days 0, 1, 3, 5, and 7). The absorbance was measured at 595 nm using a SpectraMAX M5 spectrophotometer (Molecular Devices, Sunnyvale, CA, USA).

### Soft agar colony formation assay

Soft agar colony formation assay was performed as described previously^49^. Then, cells (200 per well) were seeded in 6-well plates. After 14 days, the cells were stained with MTT and imaged. The number of colonies was counted using Colony Counter software (Keyence, Tokyo, Japan). Data are presented as mean ± SEM (*n* = 3).

### Migration assay

Cells (2.5 × 10^5^ per well) were seeded in Boyden chambers in 24-well plates (8 μm for 24-well plates; Millipore, Tokyo, Japan) and the culture medium was added into the lower chambers. After 24 h, cells were stained with crystal violet and imaged. The number of colonies was counted manually under a microscope.

### Western blot analysis

Western blot analysis was performed as described previously^49^. The antibodies used are listed in Supplementary Table S2. Immune complexes were detected using Immuno Star LD (Wako Pure Chemical Industries, Ltd, Osaka, Japan) in conjunction with a LAS-4000 image analyzer (GE Healthcare, Tokyo, Japan).

### Immunohistochemistry

IHC analysis was performed as previously described^50^. Human malignant mesothelioma tissue arrays were purchased (MS-1001a; Biomax, Houston, TX, USA). Sections were reacted with a primary antibody (CD24 antibody, 2 μg/mL; NF2 antibody, 2 μg/mL; p16 antibody, 2 μg/mL; CADM1 antibody, 2 μg/mL, Ki-67 antibody, 2 μg/mL and hematoxylin and eosin). Normal rabbit immunoglobulin G and omission of primary antibodies were used as negative controls. Immunoreactivity was independently evaluated by two investigators (S.K. and H.M.). The intensity of staining was scored as strong (3+), moderate (2+), weak (1+), or negative (0).

### Immunofluorescence

MeT-5A, HOMC-B1, DKO-MeT-5A, DKO-HOMC-B1, and Y-MESO-9 cells were cultured on glass coverslips and fixed using 4% paraformaldehyde solution for 20 min at room temperature. Cells were permeabilized with PBS containing 0.1% Triton X-100 and blocked using PBS containing 7% serum for 30 min. Then, cells were incubated with γ-catenin (desmoplakin; BD Transduction Laboratories, San Jose, CA, USA; 1:100 dilution) for 2 h at room temperature followed by fluorescence staining with anti-mouse IgG Alexa Fluor**®** 594 (Abcam, Cambridge, UK; 1:200 dilution) and Hoechst (Dojindo, Kumamoto, Japan; 1:200 dilution) for 1 h at room temperature. Images were acquired using BZ-II (Keyence) with a fluorescence microscope (BZ-X9000; Keyence).

### Statistical analysis

The statistical significance between groups was determined using one-way analysis of variance and Dunnett’s comparison. Statistical analyses were performed using SPSS 23.0 program (SPSS, Inc., Chicago, Illinois, USA). Results are expressed as mean ± SEM.

## Supplementary information

IHC analysis in MPM tissues.

Primer sets used for qRT-PCR analysis in this study.

Antibodies used in this study.

Upregulated genes in (DKO) cells

Downregulated genes in DKO cells.

Expression of CD24 in three mesothelioma subtypes.

Expression status of CD24, NF2, and p16 expression in MPM tissues.

The knockdown of CD24 reduces colony formation in the DKO cells.

Immunofluorescence analysis of desmoplakin in mesothelial and mesothelioma cells.
