# Supplementary figures and images for: Identification of CD24 as a potential diagnostic and therapeutic target for malignant pleural mesothelioma
Source: Cell Death Discov. 2020 Nov 18;6:127. doi: 10.1038/s41420-020-00364-1 (PMC7674463; doi:10.1038/s41420-020-00364-1)

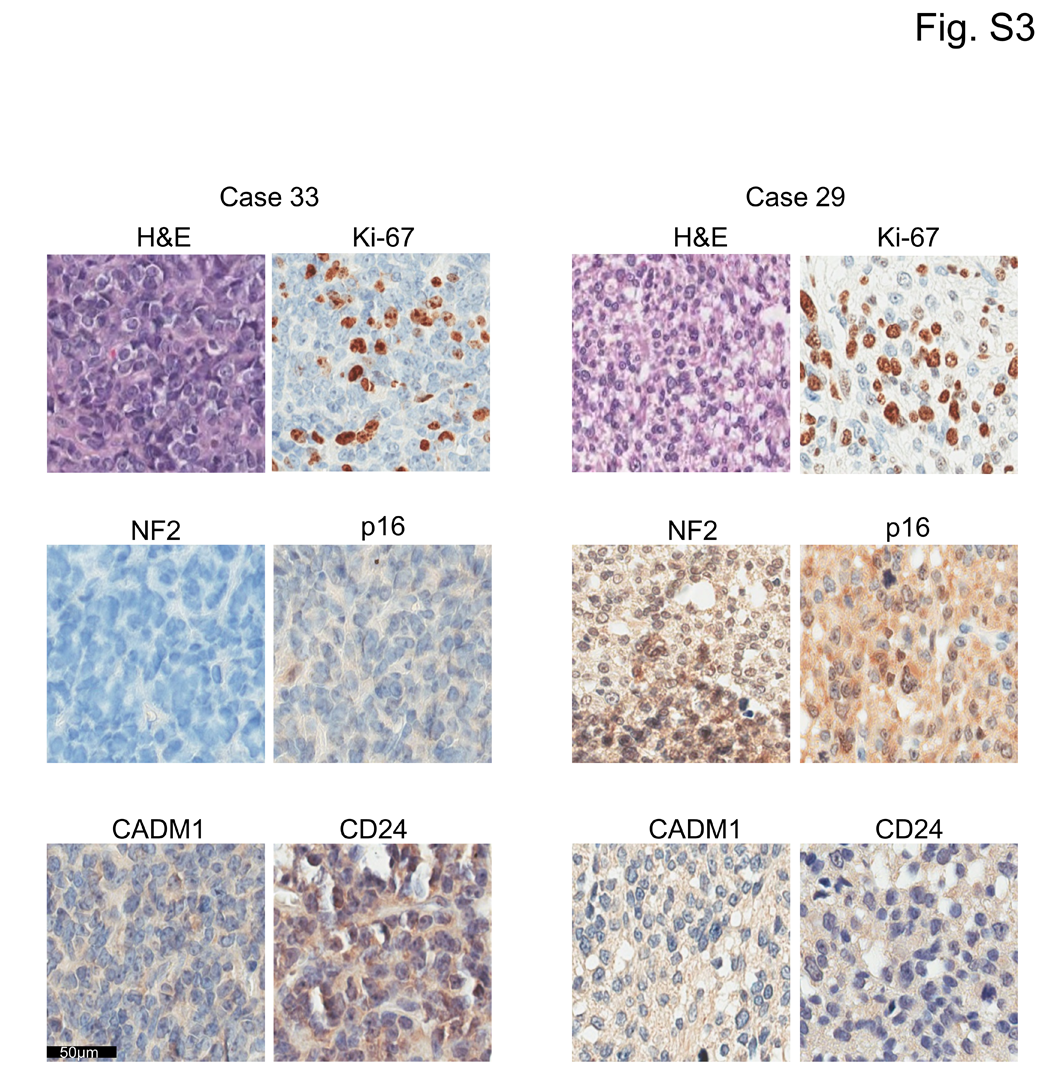

Supplement: Supplementary file 1 — IHC analysis in MPM tissues. [file 41420_2020_364_MOESM1_ESM.tif]

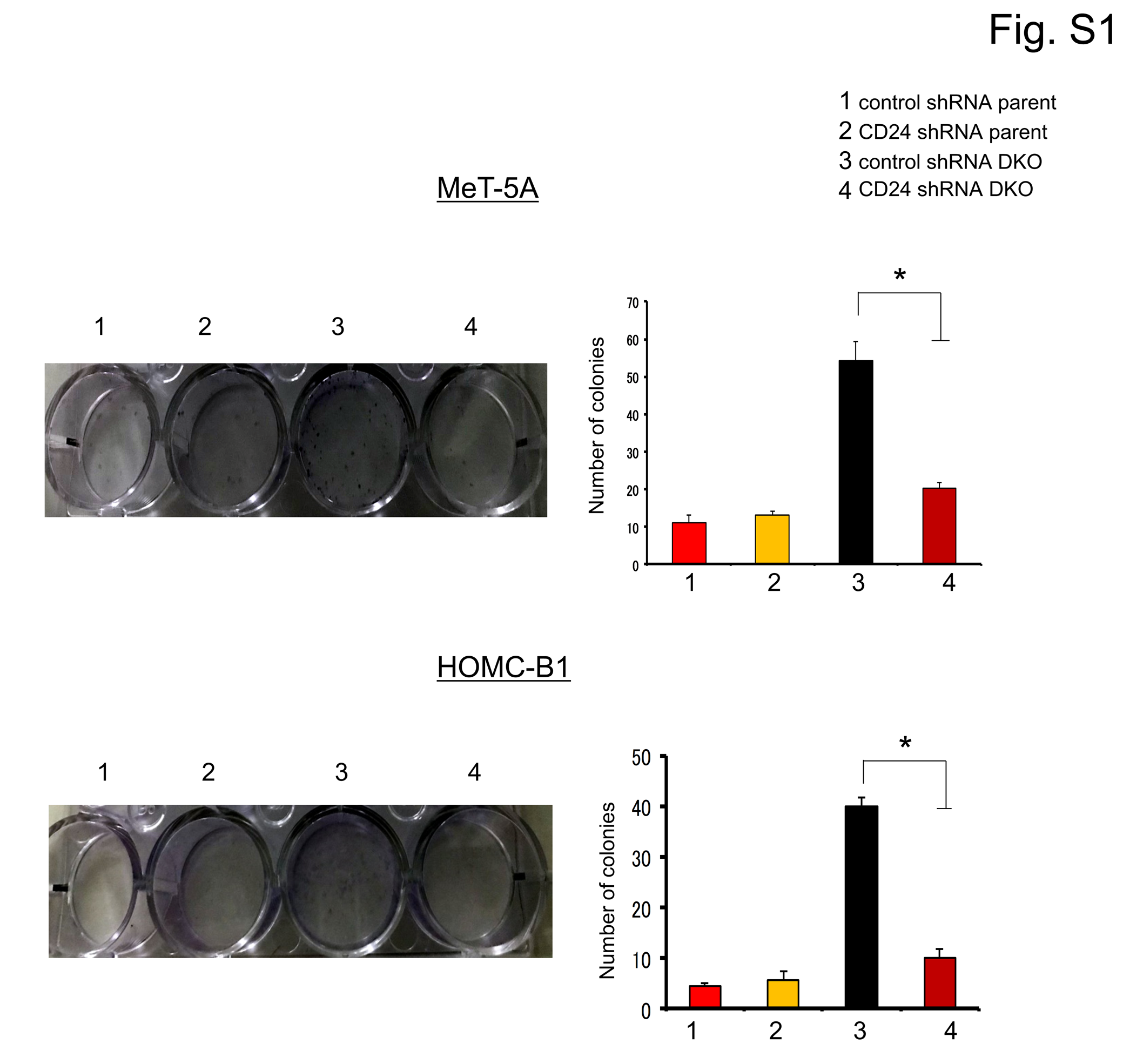

Supplement: Supplementary file 8 — The knockdown of CD24 reduces colony formation in the DKO cells. [file 41420_2020_364_MOESM8_ESM.tif]

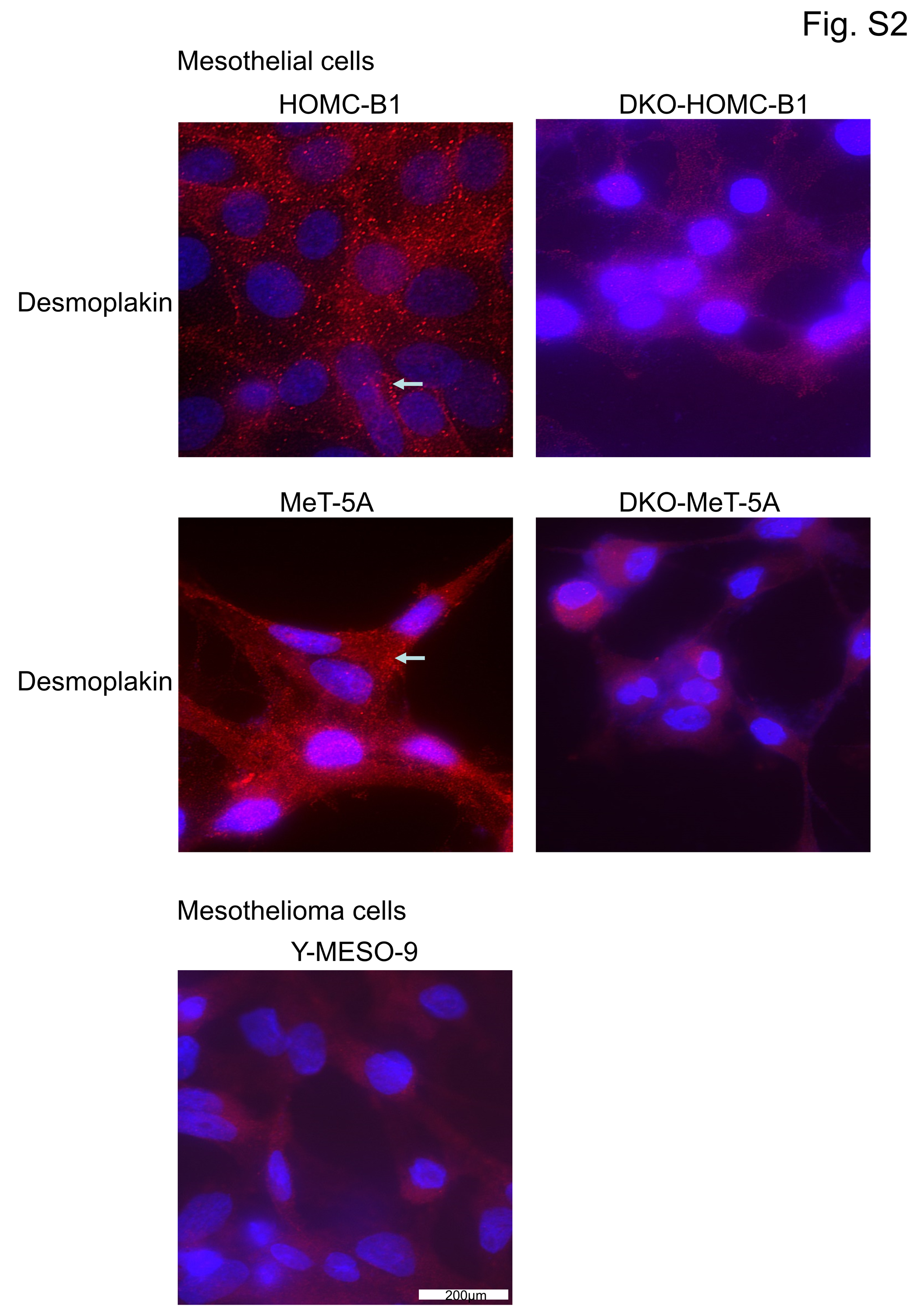

Supplement: Supplementary file 9 — Immunofluorescence analysis of desmoplakin in mesothelial and mesothelioma cells. [file 41420_2020_364_MOESM9_ESM.tif]
