# Supplementary material for: Identification of CD24 as a potential diagnostic and therapeutic target for malignant pleural mesothelioma
Source: Cell Death Discov. 2020 Nov 18;6:127. doi: 10.1038/s41420-020-00364-1 (PMC7674463; doi:10.1038/s41420-020-00364-1)
Supplement: Supplementary file 2 — Primer sets used for qRT-PCR analysis in this study. [file 41420_2020_364_MOESM2_ESM.docx]

Table S1. Primer sets used for qRT-PCR analysis in this study.

| Gene symbol | Forward prime (5'→3') | Reverse primer (5'→3') |
| --- | --- | --- |
| *PTN* | CCA GAG AGG ACG TTT CCA AC | ATCCACAGCTGCCAGTATGA |
| *CD24* | GACTCAGGCCAAGAAACGTC | CCTGTTTTTCCTTGCCACAT |
| *BMP7* | TGGTCATGAGCTTCGTCAAC | ATTGACTTGGCAACTGATGG |
| *CADM1*  *GAPDH* | CTTCTGCTGTTGCTCTTCTC  GAGTCAACGGATTTGGTCGT | GACAAGCTTCCCGTTCTCAG  GACAAGCTTCCCGTTCTCAG |
