# Supplementary material for: Identification of CD24 as a potential diagnostic and therapeutic target for malignant pleural mesothelioma
Source: Cell Death Discov. 2020 Nov 18;6:127. doi: 10.1038/s41420-020-00364-1 (PMC7674463; doi:10.1038/s41420-020-00364-1)
Supplement: Supplementary file 3 — Antibodies used in this study. [file 41420_2020_364_MOESM3_ESM.docx]

Table S2. Antibodies used in this study.

| Molecule | Cat number | Company | Species | Dilution |  |
| --- | --- | --- | --- | --- | --- |
| NF2 | SC-332 | Santa Cruz | Rb | x 200 |  |
| p16 | ab108349 | abcam | Rb | x 2000 |  |
| CD24 | ab179821 | abcam | Rb | x 1000 |  |
| CD24 | ab31622 | abcam | Rb | x 50 |  |
| Ki-67 | M724001 | DAKO | Ms | x 100 |  |
| PTN | Sc-74443 | Santa Cruz | Ms | x 200 |  |
| BMP7 | SC-53917 | Santa Cruz | Ms | x 200 |  |
| Phospho-c-Jun | #3270 | CST | Rb | x 2000 |  |
| c-JUN | #9165 | CST | Rb | x 2000 |  |
| Phospho-AKT | #4060 | CST | Rb | x 3000 |  |
| AKT | #4685 | CST | Rb | x 3000 |  |
| Phospho-70SK6 | #9234 | CST | Rb | x 1000 |  |
| p70 | #2708 | CST | Rb | x 1000 |  |
| CDK4 | #12790 | CST | Rb | x 2000 |  |
| TGF-β1 | 7666-MB-005 | R&G | Rb | x 3000 |  |
| CADM1 | CM005-3 | MBL | Chicken IgY | x 3000 | |
| p-Smad2-S250 | AP1007 | AB clonal | Rb | x 2000 |  |
| Smad2 | A7699 | AB clonal | Rb | x 2000 |  |
| Snail | SC10432 | Santa Cruz | Rb | x 2000 |  |
| E-cadherin | A11492 | AB clonal | Rb | x 2000 |  |
| N-cadherin | A0433 | AB clonal | Rb | x 2000 |  |
| Anti-rabbit IgG-HRP | #7074 | CST | Goat | x 4000 |  |
| CST, Cell Signaling Technology; Rb, rabbit; Ms, mouse | | | | |  |
