# Supplementary material for: Identification of CD24 as a potential diagnostic and therapeutic target for malignant pleural mesothelioma
Source: Cell Death Discov. 2020 Nov 18;6:127. doi: 10.1038/s41420-020-00364-1 (PMC7674463; doi:10.1038/s41420-020-00364-1)
Supplement: Supplementary file 4 — Upregulated genes in (DKO) cells [file 41420_2020_364_MOESM4_ESM.docx]

Table S3. Upregulated genes in (DKO) cells

| Gene Name | Description | Fold change* |
| --- | --- | --- |
|  |  | DKO vs parent |
| *TMSB15A* | Thymosin beta-15A | 3131.9 |
| *NCAM1* | Neural cell adhesion molecule 1 | 554.9 |
| *SYNDIG1* | Synapse differentiation-inducing gene protein 1 | 292.5 |
| *STK26* | Serine/threonine-protein kinase 26 | 287.5 |
| *LEF1* | Lymphoid enhancer-binding factor 1 | 273.1 |
| *DPYSL5* | Dihydropyrimidinase-related protein 5 | 232.0 |
| *BMP7* | Bone morphogenetic protein 7 | 190.7 |
| *CADM1* | Cell adhesion molecule 1 | 185.7 |
| *PTN* | Pleiotrophin | 120.3 |
| *LPL* | Lipoprotein lipase | 117.3 |
| *KIF1A* | Kinesin-like protein KIF1A | 110.9 |
| *SNCA* | Alpha-synuclein | 81.9 |
| *PDLIM3* | PDZ and LIM domain protein 3 | 80.2 |
| *SATB1* | DNA-binding protein SATB1 | 77.8 |
| *GALNT14* | Polypeptide N-acetylgalactosaminyltransferase 14 | 64.4 |
| *JAM2* | Junctional adhesion molecule B | 71.7 |
| *MID1IP1* | Mid1-interacting protein 1 | 64.1 |
| *TMEM108* | Transmembrane protein 108 | 63.8 |
| *GATA6* | Transcription factor GATA-6 | 58.0 |
| *B3GAT1* | Galactosylgalactosylxylosylprotein 3-beta-glucuronosyltransferase 1 | 54.4 |
| *HRASLS* | Phospholipid-metabolizing enzyme A-C1 | 50.2 |
| *FXYD6* | FXYD domain-containing ion transport regulator 6 | 43.1 |
| *HMX1* | Homeobox protein HMX1 | 37.6 |
| *ULBP1* | UL16-binding protein 1 | 27.6 |
| *EPB41L4A* | Band 4.1-like protein 4A | 25.4 |
| *SAMD5* | Sterile alpha motif domain-containing protein 5 | 22.3 |
| *CD24* | Signal transducer CD24 | 21.6 |
| *BEX2* | Protein BEX2 | 21.1 |
| *PLAG1* | Zinc finger protein PLAG1 | 20.9 |

* Upregulated genes by DKO indicated based on the differential expression (fold change) in DKO cells versus parent cells (fold change >20).
