# Supplementary material for: Identification of CD24 as a potential diagnostic and therapeutic target for malignant pleural mesothelioma
Source: Cell Death Discov. 2020 Nov 18;6:127. doi: 10.1038/s41420-020-00364-1 (PMC7674463; doi:10.1038/s41420-020-00364-1)
Supplement: Supplementary file 5 — Downregulated genes in DKO cells. [file 41420_2020_364_MOESM5_ESM.docx]

| Table S4. Downregulated genes in DKO cells. | |  |  |  | |
| --- | --- | --- | --- | --- | --- |
| Gene Name | Description | Fold change* | | | |
|  |  | DKO vs parent | | | |
| *MYL7* | Myosin regulatory light chain 2, atrial | 0.001 |  |  | |
| *ANKRD54* | Ankyrin repeat domain-containing protein 54 | 0.001 |  |  | |
| *MB21D1* | Mab-21 domain containing 1 | 0.002 |  |  | |
| *IGFBP7* | Insulin-like growth factor-binding protein 7 | 0.003 |  |  | |
| *GADD45G* | Growth arrest and DNA damage-inducible protein GADD45 gamma | 0.004 |  |  | |
| *OASL* | 2'-5'-oligoadenylate synthase-like protein | 0.004 |  |  | |
| *C3* | Complement C3 | 0.004 |  |  | |
| *PRKCDBP* | Caveolae-associated protein 3 | 0.005 |  |  | |
| *TENM2* | Teneurin-2 | 0.006 |  |  | |
| *CPNE8* | Copine-8 | 0.008 |  |  | |
| *ITGB3* | Integrin beta-3 | 0.009 |  |  | |
| *SH3RF2* | Putative E3 ubiquitin-protein ligase SH3RF2 | 0.009 |  |  | |
| *MYLPF* | Myosin regulatory light chain 2, skeletal muscle | 0.009 |  |  | |
| *CFI* | Complement factor I | 0.010 |  |  | |
| *C4B* | Complement C4-B | 0.011 |  |  | |
| *SYNPO2* | Synaptopodin-2 | 0.011 |  |  | |
| *LAMA4* | Laminin subunit alpha-4 | 0.011 |  |  | |
| *EPPK1* | Epiplakin | 0.013 |  |  | |
| *IFIT2* | Interferon-induced protein with tetratricopeptide repeats 2 | 0.013 |  |  | |
| *VAMP8* | Vesicle-associated membrane protein 8 | 0.016 |  |  | |
| *LINC00473* | Putative transcriptional regulator encoded by LINC00473 | 0.016 |  |  | |
| *TNC* | Tenascin | 0.017 |  |  | |
| *PTGFR* | Prostaglandin F2-alpha receptor | 0.018 |  |  | |
| *ABCA1* | ATP-binding cassette sub-family A member 1 | 0.018 |  |  | |
| *IFFO2* | Intermediate filament family orphan 2 | 0.018 |  |  | |
| *IFI44L* | Interferon-induced protein 44-like | 0.019 |  |  | |
| *IFI27* | Interferon alpha-inducible protein 27, mitochondrial | 0.019 |  |  | |
| *GGT5* | Glutathione hydrolase 5 | 0.021 |  |  | |
| *THBD* | Thrombomodulin-1 | 0.022 |  |  | |
| *ZNF730* | Putative zinc finger protein 730 | 0.022 |  |  | |
| *ACTG2* | Actin, gamma-enteric smooth muscle | 0.023 |  |  | |
| *ANXA8L1* | Annexin A8-like protein 1 | 0.023 |  |  | |
| *ABCC3* | Canalicular multispecific organic anion transporter 2 | 0.025 |  |  | |
| *POSTN* | Periostin | 0.026 |  |  | |
| *SLIT2* | Slit homolog 2 protein | 0.026 |  |  | |
| *FOXS1* | Forkhead box protein S1 | 0.027 |  |  | |
| *CDCP1* | CUB domain-containing protein 1 | 0.027 |  |  | |
| *THBS1* | Thrombospondin-1 | 0.028 |  |  | |
| *KAL1* | Anosmin-1 | 0.028 |  |  | |
| *GFOD1* | Glucose-fructose oxidoreductase domain-containing protein 1 | 0.028 |  |  | |
| *SNAR-A3* | small ILF3/NF90-associated RNA A3 | 0.029 |  |  | |
| *CBLN2* | Cerebellin-2 | 0.032 |  |  | |
| *LAMB3* | Laminin subunit beta-3 | 0.032 |  |  | |
| *CSRP1* | Cysteine and glycine-rich protein 1 | 0.032 |  |  | |
| *DSP* | Desmoplakin | 0.033 |  |  | |
| *OPLAH* | 5-oxoprolinase | 0.033 |  |  | |
| *NPR3* | Atrial natriuretic peptide receptor 3 | 0.034 |  |  | |
| *RSAD2* | Radical S-adenosyl methionine domain-containing protein 2 | 0.034 |  |  | |
| *TGM2* | Protein-glutamine gamma-glutamyltransferase 2 | 0.034 |  |  | |
| *BC014063* | hypothetical protein LOC151878, mRNA | 0.035 |  |  | |
| *ASAP1-IT1* | ASAP1 intronic transcript 1 | 0.035 |  |  | |
| *EFEMP1* | ERBB receptor feedback inhibitor 1 | 0.036 |  |  | |
| *NPR3* | Atrial natriuretic peptide receptor 3 | 0.036 |  |  | |
| *RELB* | Transcription factor RelB | 0.036 |  |  | |
| *CD59* | CD59 glycoprotein | 0.037 |  |  | |
| *TMEM158* | Transmembrane protein 158 | 0.037 |  |  | |
| *DSEL* | Dermatan-sulfate epimerase-like protein | 0.038 |  |  | |
| *ERRFI1* | ERBB receptor feedback inhibitor 1 | 0.039 |  |  | |
| *HMHA1* | Rho GTPase-activating protein 45 | 0.039 |  |  | |
| *IFIT3* | Interferon-induced protein with tetratricopeptide repeats 3 | 0.039 |  |  | |
| *SLC15A3* | Solute carrier family 15 member 3 | 0.040 |  |  | |
| *FN1* | Fibronectin | 0.040 |  |  | |
| *REC8* | Meiotic recombination protein REC8 homolog | 0.041 |  |  | |
| *MMP24* | Matrix metalloproteinase-24 | 0.041 |  |  | |
| *PPP1R3C* | Protein phosphatase 1 regulatory subunit 3C | 0.041 |  |  | |
| *SGK1* | Serine/threonine-protein kinase Sgk1 | 0.042 |  |  | |
| *ICAM1* | Intercellular adhesion molecule | 0.043 |  |  | |
| *CD97* | CD97 antigen | 0.044 |  |  | |
| *PLEKHA4* | Pleckstrin homology domain-containing family A member 4 | 0.044 |  |  | |
| *TXNIP* | Thioredoxin-interacting protein | 0.045 |  |  | |
| *PPP1R3B* | Protein phosphatase 1 regulatory subunit 3B | 0.046 |  |  | |
| *MEGF6* | Multiple epidermal growth factor-like domains protein 6 | 0.046 |  |  | |
| *ZNF469* | Zinc finger protein 469 | 0.047 |  |  | |
| *SH3D21* | SH3 domain-containing protein 21 | 0.048 |  |  | |
| *SPTBN1* | Spectrin beta chain, non-erythrocytic 1 | 0.048 |  |  | |
| *CD14* | Monocyte differentiation antigen CD14 | 0.050 |  |  | |
| * Downregulated genes by DKO indicated based on the differential expression (fold change) in DKO cells versus parent cells (fold change < 0.05). | | | | |  |
|  |  |  |  |  |  |
