# Supplementary material for: Identification of CD24 as a potential diagnostic and therapeutic target for malignant pleural mesothelioma
Source: Cell Death Discov. 2020 Nov 18;6:127. doi: 10.1038/s41420-020-00364-1 (PMC7674463; doi:10.1038/s41420-020-00364-1)
Supplement: Supplementary file 6 — Expression of CD24 in three mesothelioma subtypes. [file 41420_2020_364_MOESM6_ESM.docx]

Table S5. Expression of CD24 in three mesothelioma subtypes.

| Subtype Total cases CD24 (+) cases percentage (%) of  CD24 expression |
| --- |
| Epithelioid 32 29 91  Sarcomatous 10 3 33  Biphasic 3 2 66 |
