# Supplementary material for: Identification of CD24 as a potential diagnostic and therapeutic target for malignant pleural mesothelioma
Source: Cell Death Discov. 2020 Nov 18;6:127. doi: 10.1038/s41420-020-00364-1 (PMC7674463; doi:10.1038/s41420-020-00364-1)
Supplement: Supplementary file 7 — Expression status of CD24, NF2, and p16 expression in MPM tissues. [file 41420_2020_364_MOESM7_ESM.docx]

Table S6. Expression status of CD24, NF2, and p16 expression in MPM tissues.

| NF2 p16 Total cases CD24 (+) cases percentage (%) of  CD24 expression |
| --- |
| + + 19 8 42  + - 6 6 100  - - 20 20 100 |
